# Complexity of TNF-α Signaling in Heart Disease

**DOI:** 10.3390/jcm9103267

**Published:** 2020-10-12

**Authors:** Filip Rolski, Przemysław Błyszczuk

**Affiliations:** 1 Department of Clinical Immunology, Jagiellonian University Medical College, 30-663 Cracow, Poland; filip.rolski@uj.edu.pl; 2 Center of Experimental Rheumatology, Department of Rheumatology, University Hospital Zurich, 8952 Schlieren, Switzerland

**Keywords:** TNF-α, TNFR1, TNFR2, heart, cardiovascular disease, inflammation, cardiac fibrosis

## Abstract

Heart disease is a leading cause of death with unmet clinical needs for targeted treatment options. Tumor necrosis factor alpha (TNF-α) represents a master pro-inflammatory cytokine that plays an important role in many immunopathogenic processes. Anti-TNF-α therapy is widely used in treating autoimmune inflammatory disorders, but in case of patients with heart disease, this treatment was unsuccessful or even harmful. The underlying reasons remain elusive until today. This review summarizes the effects of anti-TNF-α treatment in patients with and without heart disease and describes the involvement of TNF-α signaling in a number of animal models of cardiovascular diseases. We specifically focused on the role of TNF-α in specific cardiovascular conditions and in defined cardiac cell types. Although some mechanisms, mainly in disease development, are quite well known, a comprehensive understanding of TNF-α signaling in the failing heart is still incomplete. Published data identify pathogenic and cardioprotective mechanisms of TNF-α in the affected heart and highlight the differential role of two TNF-α receptors pointing to the complexity of the TNF-α signaling. In the light of these findings, it seems that targeting the TNF-α pathway in heart disease may show therapeutic benefits, but this approach must be more specific and selectively block pathogenic mechanisms. To this aim, more research is needed to better understand the molecular mechanisms of TNF-α signaling in the failing heart.

## 1. Introduction

Heart disease refers to a group of diseases characterized by the affected function of the heart muscle. Epidemiologic data suggest that heart disease is a leading cause of death in the world. Heart failure affects 26 million people worldwide and causes 1 million annual hospitalizations in the United States and Europe [1]. Insufficient blood supply of the heart muscle by coronary arteries, which is termed coronary artery disease, represents the most common cause of heart disease. Extended ischemia in the myocardium is a life-threating condition that can lead to myocardial infarction or sudden cardiac death. Aberrant or impaired cardiac function (heart failure) can also develop in the absence of coronary artery disease. Such non-ischemic heart disease is a consequence of pathological changes in the structure of the cardiac muscle. In case of heart failure, the heart is unable to efficiently pump the blood due to ineffective muscle contraction (systolic heart failure) or relaxation (diastolic heart failure). From a clinical point of view, the type of heart failure depends on the left ventricular ejection fraction (LVEF) parameter. For LVEF < 40%, systolic heart function is impaired, and this condition is referred to as heart failure with reduced ejection fraction (HFrEF). Diastolic heart failure patients are often characterized by LVEF > 50% (or sometimes >40%); therefore, this type of heart failure is currently defined as heart failure with preserved ejection fraction (HFpEF). Both HFrEF and HFpEF patients show reduced life expectancy [2,3].

Inflammation plays an important role in the progression of many types of cardiovascular disease. On the one hand, the systemic inflammatory condition enhances atherogenesis, leading to coronary artery disease, but it also can promote the development of diastolic heart failure. On the other hand, cardiac inflammation occurs in post-ischemic myocardial events, and in more rare cases, it develops as a response to non-ischemic cardiac injury that often causes pathogenic changes in the cardiac tissue resulting in systolic dysfunction [4]. Thus, anti-inflammatory treatment has been suggested to protect the heart and cardiovascular system [5].

Tumor necrosis factor α (TNF-α) represents one of the most potent pro-inflammatory cytokines, and therefore, it was selected as the first target in the cytokine-targeted approach. Currently, TNF-α inhibitors are clinically used anti-inflammatory drugs to treat mainly patients with systemic inflammatory diseases. Roughly 1 million patients receive this type of treatment, and TNF-α antagonists are currently the most profitable class of drugs in the world, accounting for 25 billion US dollars in sales annually [6]. In case of heart failure, clinical data indicated that TNF-α inhibitors were not effective and even could worsen disease outcomes. However, the reasons of these disappointing results remain elusive.

## 2. TNF-α Biosynthesis

In humans, TNF-α is encoded by the *TNFA* gene located on chromosome 6 and shares locus with major histocompatibility complex (MHC) class II genes, which plays a central role in antigen presentation [7]. The *TNFA* gene consists of 200 nucleotide promoters with binding sites for several transcription factors, resulting in a high plasticity of transcription and responsiveness to various types of stimuli, which also vary between cell types [8].

At the post-transcript stage, the biosynthesis of TNF-α is controlled mainly through the competitive binding of the mRNA 3′ AU-rich untranslated region by RNA-binding proteins tristetraprolin (TTP) and stabilizing factor human antigen R (HuR). The dephosphorylated form of TTP effectively binds to mRNA and degrades it. The phosphorylation of TTP weakens its affinity to mRNA preventing its degradation. This allows the binding of HuR to mRNA and enables a more efficient translation of *TNFA* transcripts. Pro-inflammatory stimuli, such as lipopolysaccharide (LPS), regulate the activity of TTP and the translocation of HuR from the nucleus to cytoplasm, thereby enhancing TNF-α biosynthesis. TTP activity is regulated by p38 mitogen-activated protein kinases (MAPK), which controls TTP target genes at the post-transcriptional level and the binding of nuclear factor kappa B (NF-κB) to the promoter of TTP, which positively regulates its translation [9,10,11]. The deficiency of TTP in mice leads to increased TNF-α production that results in growth retardation, cachexia, arthritis, and autoimmune response [12]. The biosynthesis of TNF-α is regulated by a number of inflammatory mediators such as LPS, interleukin (IL)-1β, IL-6, interferon gamma (IFN-γ), tissue trauma, or hypoxia [13,14,15].

Upon translation, TNF-α is synthesized as a 17 kDa type II (i.e., possessing a single, uncleavable transmembrane segment, which anchors the protein in a cell membrane with the *C*-terminal end oriented toward cytoplasm) transmembrane protein. This membrane form of TNF-α (mTNF-α) can function as a ligand. The extracellular domain of mTNF-α can be cleaved by TNF-α cleaving enzyme (TACE; ADAM17) and released as soluble TNF-α (sTNF-α) [16]. mTNF-α and sTNF-α assemble as noncovalently bound homotrimers and in this form exert their biological functions [17].

## 3. TNF-α Receptors

**Figure 1:**
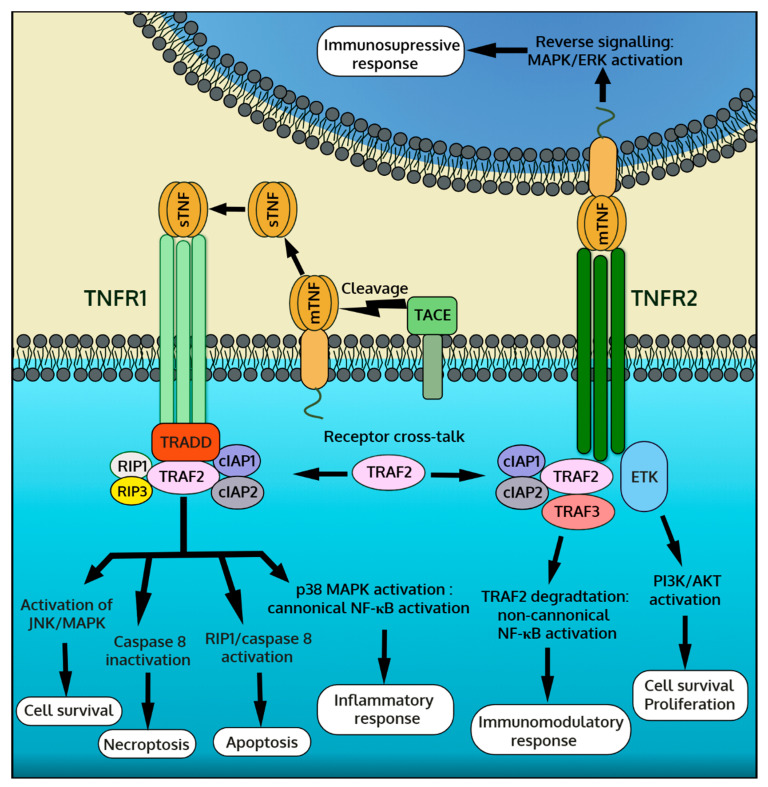
Overview of tumor necrosis factor-α (TNF-α) downstream signaling pathway mediated by two TNF-α receptors, TNFR1 and TNFR2, and by membrane form of TNF-α (mTNF-α) reverse signaling.

TNF-α represents a ligand for two types of TNF-α receptors (TNFRs), namely TNFR1 (CD120a, p55) and TNFR2 (CD120b, p75). TNFRs represent single transmembrane glycoproteins with extracellular TNF-α binding domains characterized by four tandem-repeated cysteine-rich motifs [18]. TNFRs are typically located on the cell membrane, but they can be shed and released in soluble forms with the ability to bind and neutralize the activity of circulating sTNF-α. In the body, most cells constitutively express TNFR1. In contrast, the expression of TNFR2 is often induced by pro-inflammatory factors and is restricted mainly to immune cells, but it can be also upregulated by endothelial cells or cardiomyocytes [19,20]. The activation of TNFR1 or TNFR2 depends on the bioavailability of the soluble and membrane-bound forms of TNF-α. sTNF-α shows a far greater affinity to TNFR1, whereas TNFR2 is activated mainly by mTNF-α [21]. The stimulation of TNFR1 and TNFR2 activates a distinct molecular response resulting in different effector outputs in the affected cell. Furthermore, mTNF-α is capable of transmitting reverse signaling and therefore must be considered as a receptor, too [22]. In this case, TNFRs (membrane and soluble) serve as ligands for mTNF-α. The mTNF-α reverse signaling is mainly triggered by TNFR2 [22]. A schematic presentation of TNF-α signaling is shown in Figure 1.

### 3.1. TNFR1 Signaling

In the presence of the ligand, TNFR1 recruits a number of adaptor proteins including TNFR-associated death domain (TRADD), TNF-α receptor associated factor 2 (TRAF2), receptor-interacting protein (RIP) kinase, inhibitors of apoptosis proteins (IAPs), Fas-associated death domain (FADD) and MAPK activating death domain (MADD) [19,21]. The newly formed TNFR1/TRADD/TRAF2/RIP/IAPs complex activates MAPKs, mainly c-Jun N-terminal kinase (JNK) and the p38 isoforms, and inhibitor of kappa B (IκB) kinases (IKKs). MAPKs transduce a signal into the nucleus through activation protein-1 (AP-1) and other transcription factors that bind to the specific DNA motifs of the target genes. IKKs activate the NF-κB response by degrading the IκB complex and thus releasing the p50 subunit, which translocates to the nucleus and directly regulates gene expression. These MAPK- and IKK-dependent responses contribute mainly to pro-inflammatory cytokine production and cell survival, but they also mediate other processes [19,23]. Alternatively, TNFR1 can be internalized, and due to its intracellular death domain, it can form the TNFR1/TRADD/FADD complex with pro-caspase-8. Activated caspase-8 initiates a proteolytic cascade causing cell apoptosis. Of note, TNF-α-induced apoptosis is also mediated by MAPK/JNK [24]. Furthermore, TNFR1 can induce necroptosis through mitochondrial fission. Necroptosis is independent of other caspases, and the process occurs under conditions of caspase-8 inhibitor or depletion. TNF-α mediated necrosome formation critically depends on RIP1, RIP3, and mixed lineage kinase domain like (MLKL) pseudokinase [25,26].

### 3.2. TNFR2 Signaling

Unlike TNFR1, TNFR2 lacks the intracellular death domain and is unable to bind TRADD and initiate caspase-mediated apoptosis. Instead, activated TNFR2 recruits adaptor proteins TRAF2 and IAPs, activating the canonical NF-κB signaling through IKK. In addition, TNFR2, TRAF2, and IAPs form a complex with NF-κB-inducing kinase (NIK). As a result, NIK is released from the complex and activated. Active NIK induces the non-canonical NF-κB pathway through IKKα that ultimately produces a transcriptionally active p52 subunit [27]. TNFR2 can also activate phosphatidylinositol-3-kinase (PI3K)-dependent signaling. In this pathway, activated PI3K phosphorylates protein kinase B, also known as Akt, which in turn modulates several downstream effectors [28]. TNFR2-mediated activation of the canonical and non-canonical NF-κB and PI3K/Akt pathways typically promotes cell proliferation and survival. In cells expressing both TNFRs, the cross-talk between TNFR1 and TNFR2 may occur, which is mediated by TRAF2 [19,29]. As prolonged TNFR2 activation leads to TRAF2 degradation, this negatively regulates transcription factors and the immune response but enhances TNFR1-dependent caspase-mediated apoptosis and necroptosis [21,30].

### 3.3. mTNF-α Reverse Signaling

mTNF-α acts not only as a ligand for TNFRs triggering forward signaling in the target cells but also transduces a reverse signaling back to the mTNF-α expressing cell. Physiologically, the mTNF-α reverse signaling is triggered by TNFR2 expressed by the neighboring cells [31]. Furthermore, soluble TNFRs (mainly TNFR2) or even selected anti-TNF-α antibodies can activate the mTNF-α reverse signaling [32,33]. The intracellular domain of mTNF-α shows no kinase activity; however, the binding of TNFR to mTNF-α can activate MAPKs JNK and p38 signaling and the downstream transcriptional activities in the nucleus. mTNF-α reverse signaling regulates the production of certain inflammatory cytokines, but it is also involved in the modulation of other immune processes [34]. It should be noted that the role and mechanisms of mTNF-α reverse signal transduction are not well understood.

## 4. Anti-TNF-α Therapy and Cardiovascular Diseases in Humans

In the 20th century, TNF-α has been recognized as the key pro-inflammatory cytokine in humans. This led to the development of the first cytokine-targeted therapy with etanercept approved by the Food and Drug Administration (FDA) in 1998. Anti-TNF-α therapy has revolutionized the treatment of autoimmune inflammatory diseases by offering an alternative for non-specific immunosuppressive drugs, which cause multiple adverse effects for a long-term use [35]. To date, five FDA-approved TNF-α inhibitors are being used in routine clinical practice to treat patients with rheumatoid and psoriatic arthritis, psoriasis, ankylosing spondylitis, or Crohn’s disease. TNF-α inhibitors represent the fusion protein of TNF-α receptors linked to the Fc region of human antibody (etanercept) or chimeric (infliximab), fully human (adalimumab and golimumab), or modified human (certolizumab–pegol) anti-TNF-α antibodies [36]. Although all these inhibitors neutralize TNF-α bioactivity, their therapeutic effect may vary [37,38].

The positive effects of anti-TNF-α therapy in autoimmune inflammatory diseases encouraged testing its therapeutic value in patients with systolic heart failure. These patients are characterized by elevated plasma levels of TNF-α and other pro-inflammatory cytokines [39,40,41,42], and improvement of their cardiac functions has been associated with decreasing TNF-α levels [43]. In fact, heart failure patients show also elevated cardiac TNF-α levels associated with dynamic changes in TNFR1 and TNFR2 expression [44]. Furthermore, genetic studies suggested that polymorphism in the *TNFA* gene was associated with increased risk of coronary heart disease development [45] and in case of coronary heart disease with increased risk of gastrointestinal complications [46]. Pilot studies suggested that a higher dose of etanercept increased the ejection fraction and quality-of-life scores [47,48], as well as improved systemic endothelial vasoreactivity in patients with advanced heart failure [49]. Nonetheless, randomized, double-blind, placebo-controlled studies failed to prove the therapeutic effect of etanercept in heart failure patients with reduced ejection fraction. In fact, the RECOVER (Research into Etanercept: Cytokine Antagonism in Ventricular Dysfunction) and RENAISSANCE (Randomized Etanercept North American Strategy to Study Antagonism of Cytokines) clinical trials were terminated early due to a general lack of improvement in composite clinical score and due to the dose-dependent toxicity observed in some patients [50]. Furthermore, the ATTACH (Anti-Tnf alpha Therapy Against Chronic Heart failure) study showed that high doses of infliximab in HFrEF patients increased the risk of death or heart failure-related hospitalization [51]. The injection of a single high dose of etanercept did not improve outcomes of patients following acute myocardial infarction [52]. In conclusion, continuous anti-TNF-α therapy in patients with systolic heart failure show no evident benefits and may even be harmful and exacerbate the disease. Consequently, the use of TNF-α inhibitors is not recommended for HFrEF patients.

Unlike for HFrEF patients, a long-term anti-TNF-α therapy in patients with autoimmune inflammatory diseases is generally not harmful, and it may even protect from enhanced cardiovascular complications and cardiovascular death [53,54]. Although heart failure cases have been reported in patients treated with TNF-α inhibitors [55], the risk of new onset of heart failure in patients under age 50 receiving etanercept or infliximab is low [56]. Anti-TNF-α treatment is most commonly used to treat rheumatoid arthritis. Importantly, these patients are characterized by a more rapid development of subclinical changes in diastolic function [57], and in case of incident heart failure, they are more likely to show the HFpEF phenotype [58]. In fact, rheumatoid arthritis patients with preserved left ventricular function treated with infliximab showed improvement in cardiac function [59] and decreased the left ventricular torsion [60]. A growing body of evidence suggests that anti-TNF-α therapy could effectively protect from the development of vascular diseases and atherosclerosis in particular. Standard anti-TNF-α treatment employed in treating rheumatoid arthritis has been demonstrated to decrease levels of soluble endothelial adhesions molecules [61] as well as improve arterial stiffness [62] and endothelial functions [63]. The use of TNF-α antagonists has been associated with a decreased risk of myocardial infarction [64] and development of acute coronary syndrome [65] pointing to anti-TNF-α treatment as an effective anti-atherosclerotic therapy in rheumatoid arthritis. In line with these data, large cohort clinical studies reported the unchanged or reduced overall cardiovascular-related death of rheumatoid arthritis patients receiving TNF-α inhibitors [66,67,68,69]. Importantly, in rheumatoid arthritis, anti-TNF-α therapy protects from the development of ischemic cardiac events, but it shows no cardioprotective effects in the post-ischemic heart [64]. Noteworthy, anti-TNF-α therapy in elderly rheumatoid arthritis patients might exacerbate heart failure and reduce survival [70].

Psoriasis represents another autoimmune inflammatory disease associated with increased serum levels of TNF-α [71]. Similarly to other systemic inflammatory diseases, psoriasis patients are at increased risk of developing cardiovascular diseases [72]. In psoriasis, patients treated with adalimumab showed improvement in vascular functions [73]. A large retrospective cohort study has demonstrated a significantly reduced incidence of myocardial infarction in psoriasis patients receiving TNF-α inhibitors [74]. In line with these findings, the use of TNF-α inhibitors in psoriasis lowered the occurrence of major cardiovascular events [75]. Reductions of cardiovascular events due to treatment with TNF-α inhibitors were also observed in ankylosing spondylitis and psoriatic arthritis [76,77].

Summarizing, in the light of published clinical data (summarized in the Table 1), anti-TNF-α treatment seems to reduce the risk of cardiovascular episodes mainly by inhibiting systemic inflammation and thereby suppressing the development of atherosclerosis and ischemic events. On the other hand, in the failing heart, TNF-α plays more of a cardioprotective role, but the mechanism remains unknown.

## 5. TNF-α in Animal Models of Cardiovascular Diseases

Results of anti-TNF-α treatments in patients with heart failure or with autoimmune inflammatory diseases demonstrated the relevance and a dual role of TNF-α in cardiovascular diseases in humans. However, these clinical data are insufficient to elucidate the underlaying mechanisms. Animal models of cardiovascular diseases, on the other hand, can be used to specifically define the role of gene of interest and to study cellular and molecular mechanisms. In the case of TNF-α signaling, there is a number of available transgenic mouse models allowing for the systemic or cell type-specific overexpression or genetic knockdown of selected components of the TNF-α pathway. The results of these animal studies are summarized in the Table 2. A growing body of experimental data confirmed a dual role of TNF-α and pointed to the opposing effects of TNFR1 and TNFR2. Thus, experimental data from transgenic mouse models might explain the failure of clinical application of anti-TNF-α inhibitors in heart failure patients. Prospectively, these data suggest that targeting TNFRs, rather than TNF-α, with selective agonists or antagonists might represent a more promising cardioprotective strategy in the post-ischemic heart.

### 5.1. TNF-α in Gain-of-Function Approaches

A gain-of-function approach is based on the overexpression of the gene of interest in a cell or in an organism and represents one strategy to study its function. A popular knock-in mice model non-specifically overexpressing human TNF-α shows high TNF-α production mainly in synovial fibroblasts and in endothelial cells (without evident effects in other cell types) and develops severe erosive arthritis [78,79]. Therefore, these mice are used as a mouse model of rheumatoid arthritis. Instead, transgenic mice overexpressing TNF-α in cardiomyocytes are considered as a better model to address the role of TNF-α in the heart. An initial study showed that the cardiac-restricted overexpression of TNF-α caused lethal myocarditis with diffuse lymphohistiocytic infiltrates and interstitial edema that led to cardiac death at the age of 7–11 days [80]. In contrast, in other cardiac-restricted TNF-α overexpressing models (TNF1.6 and MHCsTNF strains), most of the transgenic mice survived and developed mild inflammation and dilated cardiomyopathy phenotype with cardiac tissue remodeling as well as systolic and diastolic dysfunction associated with atrial and ventricular arrhythmias [81,82,83]. Mathematical modeling suggested that the reentrant arrhythmias spontaneously occurring in these mice are caused by a reduced intercellular coupling [84]. Yet, it needs to be noted that cardiac tissue levels of TNF-α in these models were elevated up to 200-fold comparing native myocardium. The pathogenic effect caused by cardiac-restricted TNF-α overexpression depended mainly on the TNFR1 signaling. Accordingly, left ventricular dysfunction was preserved by the adenoviral-mediated expression of soluble TNFR1 [83], and the genetic deletion of *Tnfrsf1a* (gene encoding TNFR1) improved cardiac functions and completely protected from cardiac death [85]. On the contrary, *Tnfrsf1b* (gene encoding TNFR2) genetic deficiency exacerbated heart failure and increased the lethality of TNF-α overexpressing mice pointing to the cardioprotective role of TNFR2 [85]. Interestingly, the overexpression of non-cleavable mTNF-α in cardiomyocytes led to a concentric cardiac hypertrophy phenotype without evidence of myocarditis or systolic dysfunction [86]. This may suggest that in the TNF-α overexpressing heart, the mTNF-α reverse signaling regulates different processes than TNFR1 and TNFR2.

### 5.2. Atherosclerosis and Ischemic Heart Disease Models

Atherosclerosis is a pathogenic condition of arteries characterized by the development of atherosclerotic plaques. A rupture of atherosclerotic plaques and the subsequent blood clot formation can cause life-threatening ischemic events, such as myocardial infarction or sudden cardiac death. In a typical clinical scenario of myocardial infarction, atherosclerotic plaque rupture and the subsequent thrombosis blocks blood flow in larger coronary arteries, causing an ischemic condition in the myocardium and death of cardiomyocytes. Myocardial infarct size depends on the time and the extent of ischemia as well as the subsequent inflammatory response. In animal models, myocardial infarction is typically performed in non-atherosclerotic condition.

### 5.3. Atherosclerosis

Experimental atherosclerosis is typically induced by feeding animals an atherogenic high-fat, high-cholesterol diet over at least 3 months. The development of atherosclerotic plaques is significantly enhanced in mice with naturally elevated plasma cholesterol levels, such as *Apoe^−/−^* or *Ldlr*^−/−^ mouse strains. In mice, TNF-α has been recognized as one of the most potent proatherogenic cytokines, and the formation of atherosclerotic lesions was preceded by an increased expression of TNF-α, TNFR1, and TNFR2, which were elevated even further during plaque growth [87]. Knockout of the *Tnfa* gene in *Apoe*^−/−^ mice fed a high-fat diet retarded the progression of plaque growth and decreased levels of pro-atherosclerotic factors without affecting cholesterol levels [88,89]. Experiments with the APOE*3-Leiden Tnfa^−/−^ strain fed a high-cholesterol diet also demonstrated that TNF-α promoted necrosis in plaque-infiltrating cells and enhanced advanced lesion formation [90]. Data obtained from bone marrow chimeric mice suggested that TNF-α expressed by the bone marrow cells played a key role in an *Apoe*^−/−^ mouse model of atherosclerosis [91]. In line with this data, *Apoe*^−/−^ mice on a high-fat, high-cholesterol diet receiving recombinant TNF-α developed an enhanced atherosclerotic phenotype, which could be reversed by NF-κB inhibitors [92].

In contrast to data from transgenic models, the results of the pharmacological inhibition of TNF-α in mouse models were less consistent. It has been demonstrated that recombinant soluble TNFR1 successfully attenuated the formation of aortic lesions in an *Apoe*^−/−^ model [91]. Beneficial effects of infliximab on endothelial reactive oxygen species (ROS) production and plaque formation were further confirmed in *Apoe*^−/−^ mice kept in hypoxia conditions and fed a high-fat diet [93]. However, in *Ldlr*^−/−^ mice, monotherapy with etanercept failed to reduce the development of atherosclerotic plaques, and the atheroprotective effect of etanercept was observed only in combination with cholesterol-lowering drugs [94]. Instead, treatment with anti-TNF-α monoclonal antibody CNTO5048 (neutralizing specifically murine TNF-α) surprisingly increased plaque burden, the expression of vascular inflammatory genes, and the pro-atherogenic lipid profile in hypercholesterolemic *Ldlr*^−/−^ mice [95]. These inconsistent findings could potentially be explained by the involvement of mTNF-α, because most of the anti-TNF-α antibodies neutralize exclusively sTNF-α. Indeed, the proatherogenic effect of mTNF-α has been associated with its presence on exosomes produced by dendritic cells and the activation of the pro-inflammatory NF-κB pathway in endothelial cells [96]. Although experiments with transgenic mice expressing an exclusively non-cleavable form of mTNF-α demonstrated the involvement of mTNF-α, these findings pointed primarily to the key role of sTNF-α in mouse atherogenesis [97,98,99]. On the other hand, mice with an increased expression of mTNF-α (due to a reduced expression of *Adam17*) showed enhanced macrophage adhesion and atherosclerosis in an *Ldlr*^−/−^ mouse model [98]. It should be noticed that in this model, an increased expression of mTNF-α was associated with the constitutive activation of TNFR2 signaling. Mouse models have been also used to elucidate the role of TNFR1 in the pathogenesis of atherosclerosis. *Tnfrsf1a*^−/−^ were initially reported to develop enhanced atherogenesis when fed an atherogenic diet [100]. However, *Tnfrsf1a*^−/−^ mice on a proatherogenic *Apoe*^−/−^ background showed reduced atherosclerosis, and the data pointed to the key role of TNFR1 expressed in arteries [101]. Summarizing, the proatherogenic role of TNF-α has been generally confirmed in animal studies, but there is a surprisingly high discrepancy in results obtained from different models. It seems that *Apoe*^−/−^, rather than *Ldlr*^−/−^ or non-transgenic mice, represents the most relevant animal model to study TNF-α signaling in atherosclerosis.

### 5.4. Myocardial Infarction

Experimental acute myocardial infarction is typically achieved by permanent or temporary mechanical ligation of the left anterior descending coronary artery. Animals surviving this procedure eventually develop fibrotic scars (that replaces necrotic myocardium), and their hearts show impaired function and hemodynamic abnormalities. Published data report elevated sTNF-α levels in the serum of post-infarcted mice and increased mTNF-α expression in the infarct and peri-infarct zones [102]. In a permanent occlusion model, *Tnfa*^−/−^ mice showed a significantly smaller infarct area, decreased expression of intercellular adhesion molecule 1 (ICAM-1), and lower numbers of heart-infiltrating neutrophils and macrophages [103]. However, in the same model, a lack of both TNFRs led to a significant increase in the infarction size and to an increased apoptosis of cardiomyocytes [104]. A smaller infarct size, better cardiac function, and reduced inflammatory response were observed in *Tnfa*^−/−^ mice also in a myocardial ischemia–reperfusion injury model [105]. Similarly, the blockade of TNF-α with etanercept 10 min prior to ischemia–reperfusion injury improved cardiac functions, reduced infarct size, and cardiomyocyte apoptosis [106]. Moreover, a single dose of etanercept injected at the time of myocardial infarction improved long-term cardiac function and reduced cardiac tissue remodeling in rats [107]. In another study, pharmacological inhibitor preventing TNF-α binding to its receptor (CAS1049741-03-8) reduced post-infarction inflammatory response but worsened cardiac function due to enhanced cardiomyocyte apoptosis [108]. The injection of anti-TNF-α antibody 3 h prior to ischemia–reperfusion was also shown to reduce endothelial dysfunction by reducing the production of endothelial ROS [109]. Pathogenic processes over a long term are primary mediated by TNFR1-dependent pathways, as *Tnfrsf1a*^−/−^ mice were consistently reported to develop less impaired cardiac contractile functions and showed better survival rates up to several weeks after infarction [110,111,112,113]. This phenotype was associated with the reduced expression of inflammatory cytokines, matrix metalloproteinase activity, and diminished NF-κB and MAPK activation in the cardiac tissue. Data from mice lacking an NF-κB p50 subunit confirmed the involvement of this TNFR1-downstream pathway in the pathogenesis of myocardial infarction [114]. The pathogenic role of TNFR1 in myocardial infarction is not limited to its signaling in the heart. Cardiovascular homeostasis is regulated by the subfornical organ located in the forebrain, which controls cardiac sympathetic excitation. The targeted inactivation of TNFR1 in the subfornical organ reduced left ventricular dysfunction induced by coronary artery ligation in rats [115]. Unlike TNFR1 signaling, TNFR2-dependent pathways mainly activate cardioprotective processes in the post-infarction heart. Accordingly, *Tnfrsf1b*^−/−^ mice showed exacerbated cardiomyocyte apoptosis and fibrosis as well as worsened cardiac function and long-term survival in a permanent occlusion model [110,111]. Of note, ischemia–reperfusion experiments on isolated hearts confirmed the deteriorating effect of TNF-α [116] and cardioprotective role of TNFR2 on myocardial function recovery [117]. Summarizing, mouse data underlined the activation of TNF-α signaling in the infarcted myocardium and highlighted the counteractive effects mediated by both TNFRs.

### 5.5. Non-Ischemic Heart Failure Models

Non-ischemic heart diseases refer to cardiac abnormalities occurring in the absence of coronary artery diseases. Cardiomyopathies represent the most common type of non-ischemic heart disease, in which ventricles become enlarged and stiff. Cardiomyopathies may be caused by an abnormally thick myocardium (hypertrophic cardiomyopathy) or by the dilatation of the ventricles (dilated cardiomyopathy) [118]. Hypertrophic cardiomyopathy may be genetic or can be caused by chronic hypertension or stress, and it is characterized by ineffective muscle relaxation. On the other hand, the phenotype of dilated cardiomyopathy, which is associated with left ventricular or biventricular dilatation and systolic and diastolic dysfunction, can be a consequence of the ongoing inflammatory processes in the heart. Cardiomyopathies are often progressive pathologies causing not only impaired blood pumping but also heart valve problems, blood clots, and arrhythmias, leading to heart and secondary organ failures. There is a number of established animal models that reproduce both hypertrophic and dilated cardiomyopathy conditions [119].

### 5.6. Hypertrophic Cardiomyopathy

Experimental hypertension in rodents is commonly achieved by the partial occlusion of the aorta (transverse aortic constriction model) or by continuous infusion of the vasoconstrictor angiotensin II using osmotic minipumps. In these models, increased blood pressure in the heart induces a compensatory mechanism, by which the left ventricle becomes over time thicker and thereby less effective in muscle relaxation. On the cellular level, cardiac tissue is characterized by cardiomyocyte hypertrophy and interstitial fibrosis. Data from a mouse model of pressure overload induced by aortic banding pointed to the active role of TNF-α–TNFR1 signaling in the development of hypertensive cardiomyopathy, as initially suggested by the correlation between progressive hypertrophy and increasing myocardial levels of TNF-α, TNFR1, and TACE [120,121]. In line with this suggestion, *Tnfa*^−/−^ mice developed significantly lower inflammatory response, cardiac hypertrophy, and left ventricular remodeling, showing preserved cardiac functions in several studies [120,121,122,123]. This phenotype was attributed to the abrogated production of superoxide in a TNF-α/PI3K-dependent manner in cardiomyocytes and in cardiac fibroblasts [122], changes in the expression and activity of metalloproteinases [120], decreased cardiac inflammation, and abrogated cardiomyocyte apoptosis [121]. Interestingly, in this model of hypertension, cardiac TACE activity and TNF-α levels were controlled by tissue inhibitor of metalloproteinase (TIMP)-3 [120]. The pathogenic mechanism is mainly mediated by TNFR1. Accordingly, *Tnfrsf1a*^−/−^ mice are partially protected from transverse aortic constriction-induced hypertrophy and are characterized by better survival rates [124]. In the same model, mice lacking the TNFR1 adaptor molecule TRADD also developed significantly attenuated fibrosis with better cardiac functions, suggesting a key role of the TNFR1–TRADD-dependent cell death in hypertrophic cardiomyopathy [125]. This pathogenic TNFR1 signaling seems to be counter-regulated by TNFR2. In response to the increased blood pressure induced by transverse aortic constriction, mice lacking *Tnfrsf1b* showed worsened survival rates and increased cardiac hypertrophy, and the cardioprotective TNFR2 signaling has been linked to its effects in mitochondria [124]. Moreover, mice with cardiac-specific TRAF2 deletion developed exacerbated heart failure with pathological remodeling and cardiomyocyte necroptosis [30].

Similar data were obtained in another model of hypertrophic cardiomyopathy. In an angiotensin II osmotic minipump model, *Tnfa*^−/−^ and *Tnfrsf1a*^−/−^ mice showed significantly attenuated phenotype [126,127]. In-depth analysis demonstrated reduced immunofibrotic changes in the myocardium of *Tnfrsf1a*^−/−^ mice, but there was no protective effect on diastolic dysfunction in this model [127]. In contrast, *Tnfrsf1b*^−/−^ mice receiving angiotensin II infusion developed fibrosis and showed only slight changes in expression of pro-fibrotic genes [128]. Thus, it seems that also in this model of hypertrophy, TNF-α–TNFR1 signaling is involved in disease progression.

Cardiac hypertrophy associated with diastolic dysfunction can be alternatively induced by the continuous delivery of β-adrenergic agonist isoproterenol. In this model, mice lacking TNFR1 developed reduced inflammatory response, but this was insufficient to protect them from isoproterenol-induced hypertrophy, whereas mice deficient of TNFR2 showed an increased pro-inflammatory response and exacerbated cardiac hypertrophy [129]. Of note, in vitro experiments confirmed that TNF-α indeed could enhance isoproterenol-induced cardiomyocyte hypertrophy, but surprisingly, this effect was completely blocked by anti-TNFR2 antibody [130].

Mice consuming increasing doses of ethanol over longer periods of time develop a specific type of alcoholic cardiomyopathy with fibrotic and structural changes in the left ventricle. In this model, mice showed TNFR1-dependent elevated serum levels of TNF-α, left ventricle dysfunction, and increased cardiac ROS and pro-inflammatory cytokine production [131]. Increased TNF-α levels have been also observed in a rat model of adriamycin-induced cardiomyopathy. Animals with higher serum TNF-α levels showed worse heart function and increased mortality [132].

Summarizing, data from hypertrophic cardiomyopathy models suggest that targeting TNF-α indeed might successfully prevent from disease development. It should be noted that as in other cardiovascular diseases, pathogenic TNF-α signaling is mainly mediated by TNFR1.

### 5.7. Inflammatory Heart Diseases

In animal models, heart-specific inflammation is induced either by infection with cardiotropic virus (mainly coxsackievirus B3) or by the active induction of heart-specific autoimmunity [133]. In the infectious model, myocarditis is triggered by the immune response to the virus infecting and replicating in cardiomyocytes, whereas in the autoimmune model, myocarditis is mainly mediated by the activated heart-specific CD4^+^ T lymphocytes. Published data suggest that the TNF-α–TNFR1 axis plays an active role in the development of myocarditis. In a coxsackievirus B3 model, *Tnfa*^−/−^ and *Tnfrsf1a*^−/−^ but not *Tnfrsf1b*^−/−^ mice showed strikingly reduced cardiac inflammation [134]. Interestingly, defects in TNF-α signaling showed no effect on viral titers. In a mouse model of experimental autoimmune myocarditis induced by immunization with cardiac myosin, TNF-α/β neutralizing antibodies delivered prior to but not after immunization reduced the incidence of myocarditis [135]. Furthermore, *Tnfrsf1a*^−/−^ mice were completely protected from the development of myocarditis induced by immunization with cardiac myosin or by an adoptive transfer of autoreactive T lymphocytes [136]. Although published data point to the pathogenic role of TNF-α signaling in the development of inflammatory heart disease, it should be noted that current knowledge is based on a few studies only and that the role of TNF-α signaling in the transition from myocarditis to dilated cardiomyopathy remains unknown.

**Table 2:** Summary of phenotypes observed in animal models of cardiovascular diseases in relation to modification of the TNF-α signaling pathway.

Model	Transgene/Intervention	Phenotype	Ref.
**Overexpression**			
Cardiomyocyte-specific TNF-α overexpression	none	Lethal myocarditis with interstitial edema	[80]
	none	Progressive heart failure with severe LV remodeling	[81]
	none	Calcium-dependent atrial and ventricular arrhythmias	[82]
	sTNFR1 overexpression	Preservation of LV function	[83]
	*Tnfrsf1a* ^−/−^	Improved cardiac function, reduced mortality	[85]
	*Tnfrsf1b* ^−/−^	Exacerbated heart failure, increased mortality	
Cardiomyocyte-specific mTNF-α overexpression	none	Cardiac hypertrophy without inflammation and systolic dysfunction	[86]
**Atherosclerosis**			
Atherogenic diet	*Tnfa* ^−/−^	Protection from atherosclerotic lesion formation, lowered atherogenic lipid profile, decreased IL-6 levels	[99]
	Exclusive expression of mTNF-α	Partial protection from atherosclerosis. Lower lipid deposition and macrophage accumulation, no changes in atherogenic lipid profile and IL-6	
*Apoe*^−/−^ on atherogenic diet	*Tnfa* ^−/−^	Slower plaque growth, decreased atherosclerotic markers, no changes in cholesterol levels	[88,89]
	*Tnfa* ^−/−^	Reduced plaque growth	[91]
	sTNFR1 treatment	Reduced plaque growth	
	Transplantation of *Tnfa*^−/−^ bone marrow	Reduced plaque growth	
	*Tnfrsf1a*^−/−^ grafted arteries	Reduced plaque growth and adhesion molecule expression	[101]
	Weekly infliximab injections	Improved endothelial functions, reduced atherosclerotic plaques, and decreased ROS	[93]
	Single injection of DC-derived mTNF-bearing exosomes	Increased levels of adhesion molecules in lesions, increased plaque formation	[96]
Apoe*3-Leiden on atherogenic diet	*Tnfa* ^−/−^	Higher number of early lesions and lower number of advanced lesions. Decreased necroptosis and increased apoptosis in lesion area. No changes in inflammatory parameters and lipid profiles	[90]
*Ldlr^−^*^/−^ on atherogenic diet	ADAM17 deficiency(increased mTNF and permanent TNFR2 activation)	Faster plaque growth, enhanced macrophage adhesion, increased macrophage, and smooth muscle proliferation	[98]
	Etanercept in combination with pravastatin/saprogrelate therapy	Decrease in aortic lesion area, endothelial adhesion molecules, and improved lipid profile in comparison to pravastatin/saprogrelate	[94]
	Monoclonal anti-mouse TNF-α antibody administration	Reduced plaque stability, increased vascular pro-inflammatory gene expression, and larger plaque area	[95]
	Recombinant TNF-α administration	Increased plaque burden and endothelial LDL transcytosis. Prevented by pharmacological NF-κB inhibitors	[92]
**Myocardial Infarction**			
Permanent occlusion	*Tnfa* ^−/−^	Lower infarct area, less infiltrating mononuclear cells, reduced expression of endothelial adhesion molecules at day 1 and 7	[103]
		Improved cardiac functions up to 3 days, but not at day 7	[113]
	*Tnfrsf1a* ^−/−^	Improved contractile functions, diminished hypertrophy and remodeling, reduced NF-κB activation after 4 weeks	[110]
		Lower infarct area and fibrosis, preserved cardiac functions at day 7	[113]
		Improved contractile functions, increased survival rate after 4 weeks	[111]
		Protection for infarction-induced death, improved LV functions, and decreased hypertrophy after 6 weeks	[112]
		Reduced mortality 24 h post infarction, lower inflammation, and improved cardiac recovery after 28 days	[123]
	Pharmacological TNFR1 inactivation in subfornical organ	Reduced LV dysfunction after 4 weeks	[115]
	*Tnfrsf1b* ^−/−^	Worsened remodeling, hypertrophy and contractile functions, increased fibrosis and apoptosis at day 28	[110]
		Worsened cardiac functions, increased infarct size, exacerbated fibrosis at day 3 and 7	[113]
		Exacerbated hypertrophy, fibrosis, ventricular dilatation, and dysfunction after 4 weeks	[111]
		Increased mortality during the first 7 days, reduced number of functional blood vessels in infarct area after 28 days	[123]
	Daily monoclonal anti-TNF-α antibody administration during the first week after myocardial infarction	Reduced inflammation, worsened cardiac functions, inhibited autophagy and increased apoptosis in cardiomyocytes after 1, 2, 3, and 4 weeks	[108]
	Single etanercept injection directly after myocardial infarction	Reduced inflammation, improved remodeling, and preserved LV functions after 4 days	[107]
Ischemia-reperfusion	*Tnfa* ^−/−^	Lower infarct area, improved cardiac functions, reduced cardiac NF-κB activation measured 120 min from reperfusion	[105]
	Etanercept administration 10 min prior to myocardial infarction	Lower infarct area, improved cardiac functions, 3 h, 24 h, or 14 days after reperfusion	[106]
	Anti-mouse TNF-α antibody injection 3 h prior myocardial infarction	Preserved endothelial functions, reduced endothelial production of ROS 90 min after reperfusion	[109]
**Hypertrophic Cardiomyopathy**			
Transverse aortic constriction	*Tnfa* ^−/−^	Reduced inflammatory response, decreased hypertrophy, improved cardiac functions	[121]
	*Tnfa* ^−/−^ *Timp3* ^−/−^	Attenuated LV dilation, improved cardiac functions, increased survival after 7 weeks. Complete prevention of heart disease upon additional MMP inhibitors administration	[120]
	*Tnfa* ^−/−^	Improved cardiac functions, suppression of MMPs expression, reduction in superoxide production	[122]
	*Tnfrsf1b* ^−/−^	Increased survival rates, decreased hypertrophy, improved mitochondrial functions	[124]
	*Tradd^−/−^*	Reduced hypertrophy with improved cardiac functions, attenuated TAK1/p38 MAPK phosphorylation	[125]
	*Tnfrsf1a* ^−/−^ *Tnfrsf1b* ^−/−^	Increased mortality, hypertrophy, and mitochondrial DNA damage	[124]
	*Traf2^−/−^*	TNFR1-dependent pathological remodeling and increased cardiomyocyte death	[30]
Angiotensin II-induced hypertrophy	*Tnfa* ^−/−^	Reduced hypertrophy and hypertension	[126]
	*Tnfrsf1a* ^−/−^	Slower progression of hypertrophy, reduced fibrosis and immune response	[127]
	*Tnfrsf1a* ^−/−^	No effect on early inflammatory phase, increased uptake of bone marrow-derived fibroblasts progenitors and exacerbated fibrosis	[128]
	*Tnfrsf1b* ^−/−^	No effects	[128]
Isoproterenol-induced hypertrophy	*Tnfrsf1a* ^−/−^	Reduced inflammatory response at day 1, unchanged hypertrophy at day 7	[129]
	*Tnfrsf1b* ^−/−^	Increased inflammatory response at day 1, exacerbated hypertrophy at day 7	
Alcoholic cardiomyopathy	*Tnfrsf1a* ^−/−^	Preserved LV functions, decreased ROS in LV, lower serum levels of TNF-α	[131]
**Inflammatory Heart Diseases**			
Coxsackievirus B3 induced myocarditis	*Tnfa* ^−/−^	Reduced myocarditis, no changes in virus titers	[134]
	*Tnfrsf1a* ^−/−^	Reduced myocarditis, no changes in virus titers	
	*Tnfrsf1b* ^−/−^	Unaffected myocarditis, no changes in virus titers	
Myocarditis induced by cardiac myosin immunization	Anti-TNF-α/β before immunization	Reduced myocarditis	[135]
	Anti-TNF-α/β after immunization	Unaffected myocarditis	
	*Tnfrsf1a* ^−/−^	Protection from myocarditis despite of T cell activation	[136]
Myocarditis induced by autoreactive T cell transfer	*Tnfrsf1a* ^−/−^	Protection from myocarditis	

LDL: low-density lipoprotein LV: left ventricular, MMP: matrix metalloproteinase, ROS: reactive oxygen species, *Timp3^−/−^*: metalloproteinase inhibitor 3 knockout, *Tnfa*^−/−^: tumor necrosis factor-α knockout, *Tnfrsf1a*^−/−^: TNFR1 knockout, *Tnfrsf1b*^−/−^: TNFR2 knockout, *TRADD^−/−^*: tumor necrosis factor receptor type 1-associated death domain protein knockout.

## 6. Cellular and Molecular Mechanisms of TNF-α Signaling in Cardiovascular Diseases

**Figure 2:**
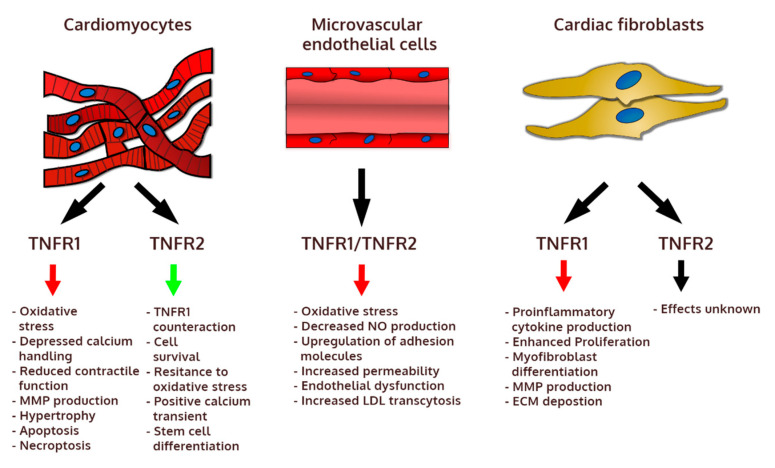
Biological effects mediated by TNFR1 and TNFR2 in main cellular components of the heart. ECM: extracellular matrix, LDL: low-density lipoprotein, MMP: matrix metalloproteinase, NO: nitric oxide.

The heart is made up of three main cell types: cardiomyocytes, cardiac microvascular endothelial cells, and cardiac stromal cells (mainly fibroblasts). In addition, heart-resident macrophages represent a small but important cell population in the healthy heart. Furthermore, in response to injury, cardiac tissue is infiltrated by immune cells, such as inflammatory monocytes and lymphocytes. It should be noted that TNF-α can be produced by many cardiac cell types, but immune cells seem to represent a particularly important source of this cytokine in cardiac pathology. TNF-α signaling controls many biological processes ranging from pro-inflammatory and proapoptotic to regenerative and cardioprotective (Figure 2). The actual effect of TNF-α depends not only on the cell type and activation of other molecular pathways, but also on expression of inducible TNFR2 (TNFR1 is generally stably expressed in nearly all cell types). As demonstrated in animal models, the deleterious effects of TNF-α are mainly mediated by the prolonged or excessive activation of TNFR1, while the activation of TNFR2 often exerts cardioprotective results.

### 6.1. Pathogenic Mechanisms

The activation of endothelial cells represents one of the best described pro-inflammatory mechanisms. Endothelial cells respond to TNF-α by an increased expression of adhesion molecules, which control the rolling and adhesion of inflammatory immune cells into the tissue. In this mechanism, both TNFRs are also critically engaged in the process of diapedesis [137]. TNF-α is also known to increase ROS levels and decrease nitric oxide production in blood vessels, which can lead to endothelial dysfunction: an initial step in atherogenesis [138]. In this process, TNF-α-induced ROS production depends on the activation of NADH oxidase [139,140]. TNF-α contributes also to the development of atherosclerotic plaques through an increase of LDL transcytosis in endothelial cells [91,92] and by regulating the activity of macrophage scavenger receptor and foam cell formation [141]. TNFR2 signaling contributes also to deleterious effects by increasing macrophage and smooth muscle proliferation [98].

In cardiomyocytes TNF-α triggers a hypertrophic response and induces apoptosis [131]. Endogenous TNF-α contributes to increased protein synthesis and hypertrophy [142] through the NF-κB-mediated production of ROS [143]. In cardiomyocytes, this process is dependent on TNFR1 activation, but it also negatively regulates calcium handling and cell contractility [144]. Moreover, TNF-α-induced superoxide production was shown to depend on NADPH oxidase activation by PI3K and to control the secretion of several matrix metalloproteinases (MMPs) [122]. Oxidative stress mediated by TNF-α is also responsible for mitochondrial DNA damage through the sphingomyelin–ceramide signaling pathway [145], which was also shown to induce apoptosis in cardiomyocytes [146]. TNFR1 promotes also cardiomyocyte apoptosis independently of NF-κB through RIP1–RIP3–MLKL axis activation by apoptosis signal-regulating kinase 1 (ASK1) [20,30].

Cardiac fibroblasts represent another heart-resident cell type that can be activated by TNF-α. In response to TNF-α, cardiac fibroblasts contribute to the inflammatory cascade by secreting monocyte chemoattractant proteins (MCP)-1 and MCP-3, which control monocyte recruitment but also positively regulate TNF-α production [147]. Furthermore, TNF-α mediates ROS production and MMP secretion in cardiac fibroblasts via the activation of PI3Kγ [122]. TNF-α contributes to hearts fibrosis also by stimulating cardiac fibroblasts proliferation and fibronectin deposition [148]. Moreover, TNF-α induces MMP9 production and promotes the transition of cardiac fibroblasts into pathogenic myofibroblasts [149]. Excessive collagen deposition and the expression of pro-fibrotic genes in these cells leading to pathological heart remodeling is controlled by TNFR1 [128,149]. TNFR1 is also important to induce fibroblast maturation from myeloid cells [150]. All these data demonstrate a wide spectrum of pathogenic effects of TNF-α in all cardiac cell types.

### 6.2. Cardioprotective Mechanisms

Most of the cardioprotective mechanisms are mediated by TNFR2. It seems that one of the most important effects of TNFR2-dependent signaling is to suppress activation of the pathogenic TNFR1 downstream pathways. It has been observed that in the absence of TNFR2, there is an increased activity of TNFR1 downstream effector molecules NF-κB [110] and MAPK p38 [129] as well as an increased production of pro-inflammatory cytokines IL-1β and IL-6 [111]. The TNFR2 cardioprotective mechanism has been described to counter-regulate the deleterious effects of TNFR1-mediated signaling in cardiomyocytes. Accordingly, the activation of TNFR2 protected cardiomyocytes from apoptosis and promoted cell cycle entry by activating endothelial/epithelial tyrosine kinase (ETK) [20], enhanced resistance to oxidative stress [130,144], and mediated positive calcium transient [144]. Of interest, TNFR2 was also shown to be critically involved in the differentiation of cardiomyocytes from stem cells in vitro [151] and in promoting cell cycle entry in resident cardiac stem cells [152]. Furthermore, TNFR2 signaling plays an important role in immunosuppression. In particularly, the activation of TNFR2 on regulatory T cells stimulated their expansion [153] and suppressed effector T cell differentiation [31]. TNFR2 signaling plays also a crucial role in the recruitment of myeloid suppressor cells [154], which exert cardioprotective functions in heart failure [155]. It should be noted that the immunomodulatory effect of TNF-α in heart failure is not well understood, mostly because of its involvement in the activation of endothelial cells and pro-inflammatory role during the early inflammatory phase.

## 7. Clinical Perspectives

TNF-α is undoubtedly an important pro-inflammatory cytokine playing a key role during the early inflammatory phase. Clinical studies and data from animal models confirmed that TNF-α enhanced the development of a number of cardiovascular pathologies. However, preventive anti-TNF-α therapy would not be recommended due to side effects and economic reasons. Instead, such targeted immunomodulatory therapeutic intervention was considered in heart failure patients, but the failure of anti-TNF-α therapy could be explained by the cardioprotective properties of TNF-α in the failing heart. Currently, our knowledge on the beneficial activity of TNF-α is limited; therefore, more experimental research is needed to uncover these processes and the underlying mechanisms. Available data suggest that most of the cardioprotective activity is mediated by TNFR2, whereas the activation of TNFR1 initiates pathogenic processes. So far, most of these findings have been obtained in mouse models using transgenic mice lacking one or the other TNFR. In the next step, targeting these receptors, either by blocking TNFR1 or activating TNFR2, should be performed pharmacologically. The development of drugs selectively targeting TNFRs, rather than blocking TNF-α activity, might represent a novel and more effective therapeutic concept in the treatment of heart disease.

## Figures and Tables

**Table 1:** Effects of anti-TNF-α treatments on cardiovascular outcomes in heart failure and in systemic inflammatory disease patients.

Type of Trial	Disease	Intervention	Outcomes	Ref.
Single-center clinical trial (18 patients)	Class III NYHA heart failure	Single infusion of etanercept (1, 4, or 10 mg/m^2^) or placebo	Dose-dependent increased quality-of-life scores and ejection fraction measured at 14th day	[47]
Single-center clinical trial (47 patients)	Class III-IV NYHA heart failure	Biweekly injections of etanercept for 3 months (5 or 12 mg/m^2^) or placebo	Dose-dependent increase in functional status, LV functions, and remodeling after 3 months	[48]
Single-center clinical trial (13 patients)	Class III NYHA heart failure	Single injection of etanercept (25 mg) in combination with standard treatment	Major improvement in systemic endothelial vasodilator capacity 7 days from intervention when compared to standard treatment group	[49]
Multi-center, double-blind clinical trial RECOVER (1123 patients)	Class II-IV NYHA heart failure	Weekly or biweekly injections of 25 mg etanercept for 6 months or placebo	Prematurely terminated due to lack of improvement in clinical outcome. No change in hospitalization or death occurrence	[50]
Multi-center, double-blind clinical trial RENAISSANCE (925 patients)	Class II-IV NYHA heart failure	Biweekly or 3 times a week injections of 25 mg etanercept for 6 months or placebo	Prematurely terminated due to lack of improvement in clinical outcome. Worsened clinical composite score in some patients	[50]
Multi-center, double-blind clinical trial ATTACH (150 patients)	Class III-IV NYHA heart failure	Injections of 5 or 10 mg/kg infliximab at 0, 2, and 6 weeks or placebo	After 14 weeks modest improvement in ejection fraction at 5 mg/kg dose. No improvement in composite clinical score at either dose, increased hospitalization and death occurrence at 10 mg/kg dose	[51]
Randomized controlled trial (26 patients)	Acute myocardial infarction	Single infusion of 10 mg etanercept in combination with standard treatment	Reduced systemic inflammation, increased platelet activation. No effect on peripheral vasomotor or fibrinolytic function when compared to standard treatment	[52]
Single-center clinical trial (23 patients)	Rheumatoid arthritis	3 mg/kg infliximab infusions every 2 months	Improvement in LV ejection fraction, reduction in IL-6, endothelin 1, and NT-proBNP serum levels	[59]
Single-center clinical trial (68 patients)	Rheumatoid arthritis	180 days of infliximab or prednisolone treatment	Improvement in LV longitudinal and radial systolic deformation and decreased LV torsion in comparison to prednisolone treatment	[60]
Multi-center comparative study (14,258 patients)	Rheumatoid arthritis	90+ days of adalimumab, etanercept, or infliximab treatment with median of 5 years follow-up	Significantly decreased risk of myocardial infarction in comparison to patients receiving synthetic DMARD	[64]
Multi-center comparative study (7704 patients)	Rheumatoid arthritis	Long-term treatment (average of 2 years) with adalimumab, etanercept, and infliximab	Significantly decreased risk of acute coronary syndrome compared to biologic-naïve RA patients or DMARD treatment	[65]
Multi-center comparative study (10,156 patients)	Rheumatoid arthritis	Long-term treatment (median exposure period of 22,9 months) with adalimumab, etanercept, and infliximab	Reduced risk of cardiovascular-related death compared to patients receiving DMARD	[66]
Multi-center retrospective comparative study (20,811 patients)	Rheumatoid arthritis	Long-term treatment (median duration of 20 months) with TNF-α inhibitors	No change in cardiovascular-related death risk, improved cardiovascular outcomes in younger patients	[67]
Multi-center retrospective comparative study (7077 patients)	Rheumatoid arthritis	Long term (up to 5 years) treatment with TNF-α inhibitors	Reduction in cardiovascular-related death in women	[68]
Multi-center retrospective comparative study (20,243 patients)	Rheumatoid arthritis	Switch from non-biological disease modifying antirheumatic drug to TNF antagonists	No change in risk of cardiovascular event (including in patients with heart failure history)	[69]
Multi-center retrospective comparative study (7077 patients)	Rheumatoid arthritis	Long-term treatment (1–2 years) with TNF-α inhibitors or methotrexate	Increased risk of heart failure onset and exacerbation of existent heart failure in elderly patients treated with TNF-α inhibitors	[70]
Multi-center comparative study (8845 patients)	Psoriasis	At least 2 months of adalimumab, etanercept, or infliximab treatment	Reduced risk of myocardial infarction in comparison to TNF-α inhibitor naïve patients	[74]
Multi-center comparative study (17,729)	Psoriasis	150 days of adalimumab, etanercept, infliximab, or methotrexate treatment	Treatment with TNF-α inhibitors was shown to reduce overall cardiovascular event risk in comparison to methotrexate	[75]
Multi-center retrospective comparative study (4140 patients)	Rheumatoid arthritis, psoriasis, ankylosing spondylitis	Long-term treatment (at least 1 year) with TNF-α inhibitors	Continuous use of TNF-α inhibitors reduced the incidence of major cardiovascular events in comparable manner in all studied diseases	[76]
Multi-center comparative study (319)	Psoriatic arthritis	Long-term treatment (2–3 years) with TNF-α inhibitors	Reduced atherosclerosis in men, but not women, receiving TNF-α inhibitors. Reduction of vascular inflammation in both sexes	[77]

LV: left ventricle, NT-proBNP: *N*-terminal prohormone of brain natriuretic peptide, DMARD-disease-modifying antirheumatic drugs.
